# Reduction of the Influence of Laser Beam Directional Dithering in a Laser Triangulation Displacement Probe

**DOI:** 10.3390/s17051126

**Published:** 2017-05-15

**Authors:** Hongwei Yang, Wei Tao, Zhengqi Zhang, Siwei Zhao, Xiaoqia Yin, Hui Zhao

**Affiliations:** Department of Instrument and Engineering, Shanghai Jiao Tong University, Shanghai 200240, China; yanghongwei@sjtu.edu.cn (H.Y.); zhengqi.zhang0611@gmail.com (Z.Z.); zsw122@sjtu.edu.cn (S.Z.); xiaoq1216@sjtu.edu.cn (X.Y.); huizhao@sjtu.edu.cn (H.Z.)

**Keywords:** laser triangulation displacement probe, laser beam pointing, prism

## Abstract

Directional dithering of a laser beam potentially limits the detection accuracy of a laser triangulation displacement probe. A theoretical analysis indicates that the measurement accuracy will linearly decrease as the laser dithering angle increases. To suppress laser dithering, a scheme for reduction of the influence of laser beam directional dithering in a laser triangulation displacement probe, which consists of a collimated red laser, a laser beam pointing control setup, a receiver lens, and a charge-coupled device, is proposed in this paper. The laser beam pointing control setup is inserted into the source laser beam and the measured object and can separate the source laser beam into two symmetrical laser beams. Hence, at the angle at which the source laser beam dithers, the positional averages of the two laser spots are equal and opposite. Moreover, a virtual linear function method is used to maintain a stable average of the positions of the two spots on the imaging side. Experimental results indicate that with laser beam pointing control, the estimated standard deviation of the fitting error decreases from 0.3531 mm to 0.0100 mm, the repeatability accuracy can be lowered from ±7 mm to ±5 μm, and the nonlinear error can be reduced from ±6 % FS (full scale) to ±0.16 % FS.

## 1. Introduction

**Figure 1:**
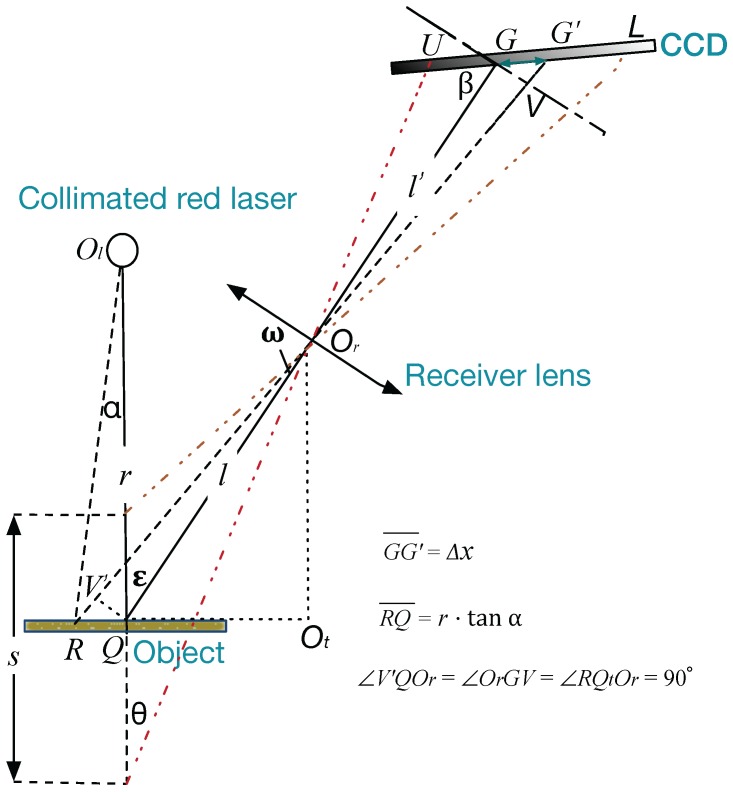
Structure of the optical path when the laser beam is dithered by an angle α.

Laser triangulation displacement probes (LTDPs) have been widely used for industrial detection [1,2,3] because of their noncontact and high-precision properties. The principles of an LTDP are illustrated in Figure 1. A collimated laser beam projects a laser dot onto the measured object. Then, the diffused laser light is collected by a receiver lens, and a dot is imaged on a charge-coupled device (CCD). When the object dot moves in a direction perpendicular to the optical axis of the laser, a corresponding displacement will occur for the image dot on the CCD.

As shown in Figure 1, ε is defined as the observed angle between the source laser beam and the optical axis of the receiver lens, and β is defined as the image angle between the CCD and the optical axis. ε and β must satisfy the Scheimpflug condition [4]. *S* is the distance over which the object moves, and UL is the corresponding image distance. If *s* is upward along the optical axis, the sign is “+”; otherwise, it is “-” . The relationship between *s* and UL is

(1) $$UL=\frac{sl^{\prime }\times \sin\left(\beta \right)}{l\sin(\beta )\mp s\sin(\varepsilon +\beta )}$$

The measurement accuracy of the LTDP is affected by the speckle [5], the color of the measured object [6], the surface texture [7], the ambient light, variation in the laser beam intensity [6,8], distortion of the receiver lens [9], and instability of the source laser beam. Shen et al. [8] introduced a digital correlation method for suppressing the speckle noise. The results showed that the measurement range reached 1 μm, and the experimental errors were reduced below 2 %. Oh et al. [10] improved the hardware structure by inserting a diffraction grating between the receiver lens and the CCD. The diffraction grating simultaneously generated −1—and 0—order. light intensity distributions on the CCD. This method can reduce the measurement time by averaging the results of the two orders. Similarly, Blais [11] and Loranger [12] introduced a Biris method by inserting a dual-aperture mask next to the imaging lens to create two points on the CCD. Jung et al. [6] and Shen et al. [8] reported an adaptive control technique to maintain a stable beam intensity. Keyence [13] proposed a real peak detection algorithm that aimed to detect the true peak position value rather than the traditional centroid value to avoid the effect of the oversized diameter. Zbontar et al. [14] introduced a double curve fitting algorithm to compensate the skewed distribution. In addition, they [14] used an ultraviolet (UV) laser to improve the required signal quality. However, the UV laser will induce photochemical effects, which might lead to material degeneration; thus, this method is only used to detect certain materials such as high-end lenses or hot metals.

In this paper, an LTDP that uses a laser beam pointing control setup (LPC) is proposed to decrease the effect of directional dithering of a laser beam. This probe simultaneously generates two symmetrical laser intensity distributions. Since the averages of the two positions on the detected surface are constant, the influence of laser dithering can be avoided. Moreover, the speckle noise related to the measured surface roughness and stray light can be reduced because the two measurement results are averaged.

The remainder of the paper is arranged as follows. We analyze the effect of laser dithering on the measurement accuracy of an LTDP in Section 2. Section 3 describes the LTDP with the LPC and introduces the laser dithering compensation algorithm. Experiments for verifying the performance of the probe are presented in Section 4. Finally, the conclusions are summarized in Section 5.

## 2. Effect of the Laser Beam Directivity on the Measurement Accuracy

**Figure 2:**
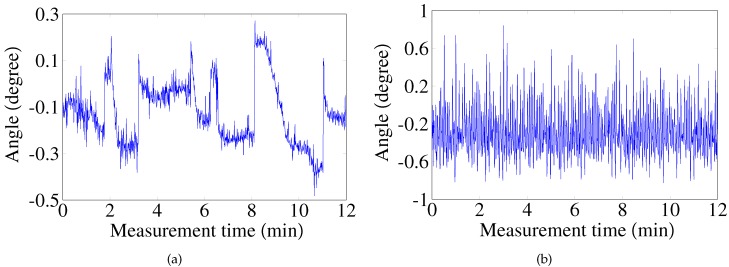
Laser beam pointing images captured by a beam profiler (BP100, Thorlabs., Newton, NJ, USA): (a) Green laser. λ = 520 nm, and the power is 4.6 mW; (b) Red laser. λ = 635 nm, and the power is 2.1 mW.

Figure 2a,b show two different occurrences of laser dithering produced by two different collimated lasers manufactured (Xi’an Minghui Optoelectronic Technology Co., Ltd., Xi’an, China) The experimental results show that the laser dithering angle is usually within (-1°, 1°).

The relationship between the dithering angle and the imaging error is deduced as follows. As shown in Figure 1, a stable pointing laser source emits the light spot *Q* on an object. The diffused light is collected by a receiver lens, and the light spot *G* is imaged on the CCD. When the source laser beam is dithered by the angle α, the light spot *R* is projected onto the object. Then, the diffused light is collected, and the light point $G^{\prime }$ is imaged on the CCD. The angle between the rays $O_{l}Q$ and $O_{l}R$ is defined as α, and the angle between the rays $RO_{r}$ and $QO_{r}$ is defined as ω. Moreover, the auxiliary line GV, which is perpendicular to the ray QG, and the auxiliary line $QV^{\prime }$, which is perpendicular to the ray QG, are added separately. Thus $∠V^{\prime }QO_{r}=∠O_{r}GV=90^{\circ }$.

$∠V^{\prime }RQ=90^{\circ }-\left(\omega +∠QO_{r}O_{t}\right)=90^{\circ }-\omega -\varepsilon$; considering $▵RV^{\prime }Q$ in Figure 1, by the law of sines,

(2) $$\frac{\bar{QR}}{\sin(90^{\circ }\;+\;\omega )}=\frac{\bar{QV^{{}^{\prime }}}}{\sin(90^{\circ }\;-\;\omega \;-\;\varepsilon )}\Rightarrow \bar{QV^{{}^{\prime }}}=\frac{\cos(\omega \;+\;\varepsilon )}{\cos\omega }\bar{QR}$$

Since $▵V^{\prime }QO_{r}$∼$▵GVO_{r}$, $\bar{QV^{\prime }}$ is expressed as follows:

(3) $$\bar{GV}=\frac{l^{{}^{\prime }}}{l}\bar{QV^{{}^{\prime }}}$$

where *l* is the length of $\bar{QO_{r}}$, and $l^{\prime }$ is the length of $\bar{O_{r}G}$. Using Equation (2),

(4) $$\bar{GV}=\frac{l^{{}^{\prime }}}{l}\frac{\cos(\omega +\varepsilon )}{\cos\omega }\bar{QR}$$

In ▵ $O_{r}O_{t}R$, $\omega +\varepsilon =\tan^{-1}\left(RO_{t}/O_{r}O_{t}\right)$. In ▵ $O_{l}RQ$, $\bar{QR}=r\times \tan\alpha$; thus, ω is expressed as follows:

(5) $$\omega =\tan^{-1}\frac{\bar{RO_{t}}}{\bar{O_{r}O_{t}}}-\varepsilon =\tan^{-1}\frac{r\;\cdot \;\tan\alpha +l\;\cdot \;\sin\varepsilon }{l\;\cdot \;\cos\varepsilon }-\varepsilon =Z-\varepsilon$$

where $Z=\tan^{-1}\left(r\;\cdot \;\tan\alpha +l\;\cdot \;\sin\varepsilon \right)/\left(l\;\cdot \;\cos\varepsilon \right)$. Combining Equations (4) and (5), $\bar{GV}$ is expressed as follows:

(6) $$\bar{GV}=T\;\cdot \;r\;\cdot \;\tan\alpha \;\cdot \;\frac{\cos Z}{\cos(Z-\varepsilon )}$$

where $T=l/l^{\prime }$.

Considering the inclination angle β of the CCD, the relationship between the dithering angle α and the dithering error ▵x over the distance $GG^{\prime }$ on the image side can be expressed as follows:

(7) $$▵x=T\times r\times \tan\alpha \times \frac{\cos Z}{\cos(Z-\varepsilon )}\times \frac{\cos\omega }{\sin(\beta -Z+\varepsilon )}$$

As shown in Equation (7), the relationship between ▵x and α is approximately linear. When $l^{\prime }=59\mathrm{mm}$, l=68.5mm, r=60mm, ε=0.349, β=0.5916, and α∈(−1°,1°), the slope is approximately 1.6350. Thus, when the source laser beam is dithered by an angle of -1° to 1°, the measurement accuracy will decrease approximately linearly as the dithering angle increases.

## 3. Measurement Methodology

### 3.1. Basic Layout

**Figure 3:**
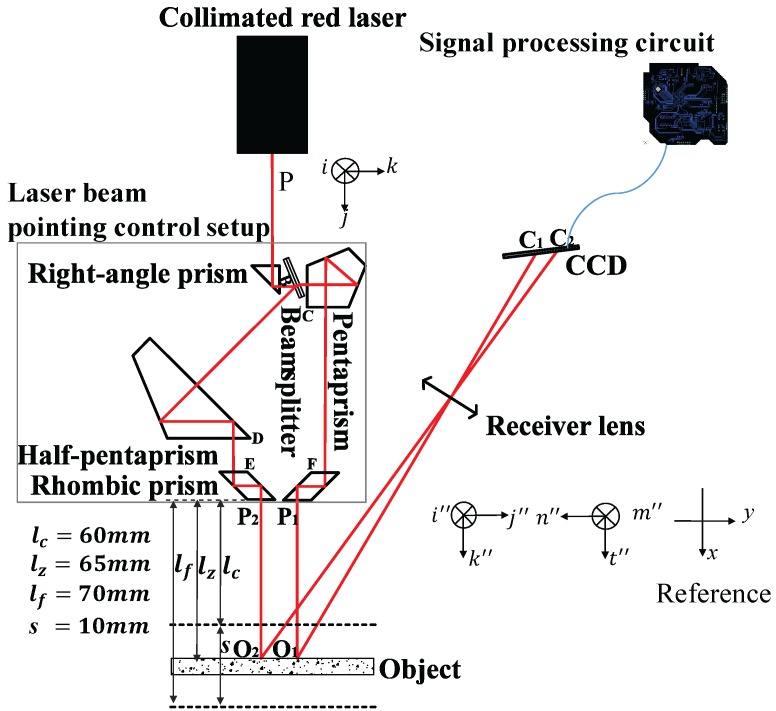
Optical structure of the scheme for reduction of the influence of laser beam directional dithering in laser triangulation displacement probe.

To overcome the effect of laser dithering, several methods have been proposed. In general, these methods utilize feedback control achieved by using an error or a reference signal. N. Zhavoronkov [15] reported a long-term femtosecond laser beam stabilization system that consisted of a tilting mirror system and a CCD camera. Ajai Kumar [16] introduced a fuzzy control setup comprising a high-resolution monochrome CCD camera, a piezoelectrically driven mirror, and a fuzzy logic toolkit LabVIEW. Newport [17] introduced a collimated model of the laser feedback, which realized laser beam tracing and stability control by adjusting two fast steering mirrors. However, these methods are inappropriate for an LTDP because the structures mentioned in these studies have a large volume and are not suitable for a single shot system and there is a dependence on the error or reference signal. Francois Blais [11] and Soichi Ibaraki [18] reported a dual-view triangulation method. This structure can suppress the influence of the detected surface, but cannot address the source laser beam directional dithering. Therefore, a scheme for reduction of the influence of laser beam directional dithering in a LTDP is designed in this study, which consists of a collimated red laser, a laser beam pointing control setup (LPC), a receiver lens, and a CCD, as shown in Figure 3.

The LPC consists of a right-angle prism, a beam splitter, a pentaprism, a half-pentaprism, and two rhombic prisms. The angle between the beam splitter and the optic axis of the collimated red laser is 22.5°. The collimated red laser beam is reflected by the right-angle prism and split into two laser beams by the beam splitter. One laser beam is reflected four times by the pentaprism and rhombic prism, and the laser beam $P_{1}$ is formed. Another laser beam is reflected four times by the half-pentaprism and another rhombic prism, and the laser beam $P_{2}$ is formed. The two rhombic prisms can shorten the distance traveled by the two laser beams, which are located at positions perpendicular to the optical axes of $P_{1}$ and $P_{2}$.

With respect to the LPC, the closer point, zero point and farther point were set at distances of 60 mm, 65 mm and 70 mm, respectively, with an error of 0.2 mm.

(i,j,k) is defined as the unit vector of the source laser beam *P*, and $\left(i^{\prime \prime },j^{\prime \prime },k^{\prime \prime }\right)$ is the unit vector of $P_{1}$. According to the prism turning theorem, the interaction matrix $B_{1}$ of $P_{1}$ is

(8) $$B_{1}=\left[\begin{array}{ccc} 1 & 0 & 0\\ 0 & -1 & 0\\ 0 & 0 & 1 \end{array}\right]$$

Furthermore, $\left(m^{\prime \prime },n^{\prime \prime },t^{\prime \prime }\right)$ is the unit vector of $P_{2}$, and the interaction matrix $B_{2}$ of $P_{2}$ is

(9) $$B_{2}=\left[\begin{array}{ccc} 1 & 0 & 0\\ 0 & 1 & 0\\ 0 & 0 & 1 \end{array}\right]$$

Comparing Equations (8) and (9), the basis vector $j^{\prime \prime }$ of $B_{1}$ and $n^{\prime \prime }$ of $B_{2}$ are opposite, which means that the directions of $P_{1}$ and $P_{2}$ are opposite.

To ensure that the positional variations in $O_{1}$ and $O_{2}$ are equal, the positional relationship among the right-angle prism, pentaprism, half-pentaprism, and rhombic prisms must satisfy

(10) $$\begin{array}{c} |x_{1}+x_{3}-x_{2}+y_{3}|=\sqrt{2L^{2}+h^{2}-2Lh\left[\cos\left(22.5^{\circ }\right)-\sin\left(22.5^{\circ }\right)\right]}\\ l_{1}=2l_{2}\\ x_{E}=x_{F} \end{array}$$

where $L={\left[x_{1}-y_{1}+y_{2}-x_{2}-\sqrt{2}\;\cdot \;\left(x_{1}+y_{1}\right)\right]}^{1/2}$, ($x_{1},y_{1}$) are the coordinates of point B, ($x_{2},y_{2}$) are the coordinates of point D, ($x_{3},y_{3}$) are the coordinates of point C, 2h is the thickness of the beam splitter, $l_{1}$ is the length of the short side at 45° in the half-pentaprism, $l_{2}$ is the length of the side at 90° in the pentaprism, $x_{E}$ is the *x* coordinate of point E, and $x_{F}$ is the *x* coordinate of point F. In summary, if the positional relationship of the prisms satisfy Equation (10), the average value of $O_{1}$ and $O_{2}$ remains invariant.

### 3.2. Laser Dithering Compensation Algorithm

**Figure 4:**
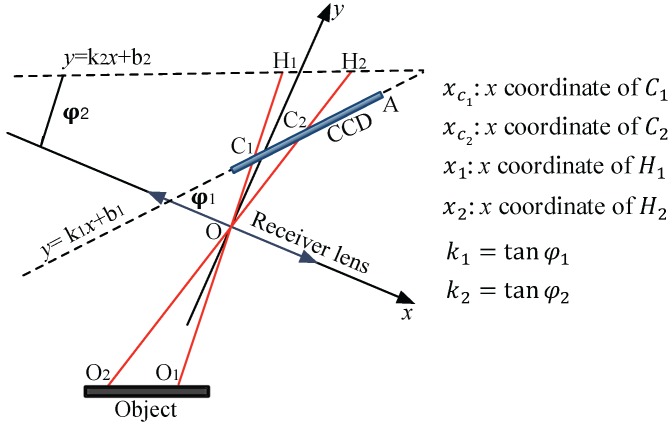
Working principle of the laser dithering compensation algorithm.

As shown in Figure 4, a coordinate system (Oxy) is constructed, where *O* is the point of the receiver lens, and the *x* axis is coincident with the plane of the receiver lens. The linear function of the CCD is defined as $y=k_{1}x+b_{1}$, where $k_{1}=\tan\varphi_{1}$. Point *A* is the position at which the pixel value on the CCD is zero.

The laser dithering compensation algorithm is described as follows. First, the peak value $y_{P_{1}}$ and the corresponding pixel value $x_{P_{1}}$ of the point $C_{1}$ as well as the peak value $y_{P_{2}}$ and the corresponding pixel value $x_{P_{2}}$ of the point $C_{2}$ are determined. Then, the centroids $C_{d_{1}}$ and $C_{d_{2}}$ of $C_{1}$ and $C_{2}$, respectively, are calculated using

(11) $$\begin{array}{c} C_{d_{1}}=\frac{\sum_{i=x_{P_{1}}-w}^{x_{P_{1}}-1}x_{i}\;\cdot \;y_{i}+x_{P_{1}}\;\cdot \;y_{P_{1}}+\sum_{j=x_{P_{1}}+1}^{x_{P_{1}}+w}x_{j}\;\cdot \;y_{j}}{\sum_{i=x_{P_{1}}-w}^{x_{P_{1}}-1}y_{i}+y_{P_{1}}+\sum_{j=x_{P_{1}}+1}^{x_{P_{1}}+w}y_{j}}\\ C_{d_{2}}=\frac{\sum_{p=x_{P_{2}}-w}^{x_{P_{2}}-1}x_{p}\;\cdot \;y_{p}+x_{P_{2}}\;\cdot \;y_{P_{2}}+\sum_{q=x_{P_{2}}+1}^{x_{P_{2}}+w}x_{q}\;\cdot \;y_{q}}{\sum_{i=x_{P_{2}}-w}^{x_{P_{2}}-1}y_{q}+y_{P_{2}}+\sum_{q=x_{P_{2}}+1}^{x_{P_{2}}+w}y_{q}} \end{array}$$

where *w* is the data width, *i* is the pixel value before $x_{P_{1}}$, *j* is the pixel value after $x_{P_{1}}$, *p* is the pixel value before $x_{P_{2}}$, and *q* is the pixel value after $x_{P_{2}}$.

The *x* coordinates of $C_{1}$ and $C_{2}$ in the (Oxy) system are then expressed as follows:

(12) $$\begin{array}{c} x_{C_{1}}=x_{A}-t\;\cdot \;C_{d_{1}}\;\cdot \;\cos\varphi_{1}\\ x_{C_{2}}=x_{A}-t\;\cdot \;C_{d_{2}}\;\cdot \;\cos\varphi_{1} \end{array}$$

where $x_{A}$ is the *x* coordinate of point A, and *t* is the resolution of the CCD.

Since the CCD is not parallel to the plane of the object but forms an angle $\varphi_{1}$, the average position (AVG) of $x_{C_{1}}$ and $x_{C_{2}}$ is not appropriate for calibration. Here, we construct a virtual calibrated line $y=k_{2}x+b_{2}$, which is parallel to the plane of the object, as shown in Figure 4, where $k_{2}=\tan\varphi_{2}$.

The *x* coordinate $x_{1}$ of the point $H_{1}$ and the *x* coordinate $x_{2}$ of the point $H_{2}$ are

(13) $$\begin{array}{c} x_{1}=\frac{x_{C_{1}}\;\cdot \;b_{2}}{\left(k_{1}-k_{2}\right)\;\cdot \;x_{C_{1}}+b_{1}}\\ x_{2}=\frac{x_{C_{2}}\;\cdot \;b_{2}}{\left(k_{1}-k_{2}\right)\;\cdot \;x_{C_{2}}+b_{1}} \end{array}$$

As a result, AVG of $x_{1}$ and $x_{2}$ is

(14) $$AVG=\frac{x_{1}+x_{2}}{2}$$

Since the positional variations in $O_{1}$ and $O_{2}$ are equal and opposite, the value of AVG remains constant.

## 4. Experimental Test

### 4.1. Presetting

**Figure 5:**
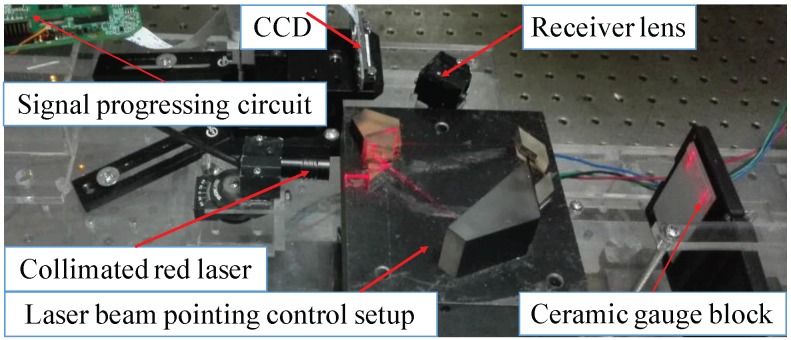
Reduction of the influence of laser beam directional dithering in a laser triangulation displacement probe.

An LTDP with an LPC is shown in Figure 5, where the LPC is inserted between the collimated red laser and the detected object. The main devices used in this system are shown in Table 1.

According to the design parameters of the LTDP, the linear function of the CCD is y=0.6009x+46.09, and the virtual calibrated line is y=0.6745x+46.09.

### 4.2. Verification of the Laser Dithering Compensation Algorithm

**Figure 6:**
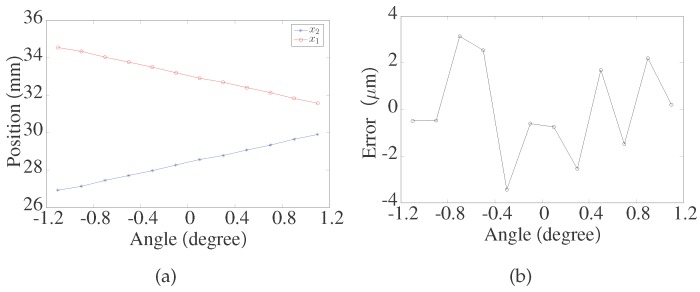
(**a**) Values of $x_{1}$ and $x_{2}$; (**b**) Extreme error of AVG.

Figure 6 shows the values of $x_{1}$ and $x_{2}$ when the ceramic gauge block is located at one fixed position, and the source laser beam *P* is rotated within ±1.1° with an increment of 0.2°. As shown in Figure 6a, the variations in $x_{1}$ and $x_{2}$ are equal and opposite. As shown in Figure 6b, the extreme error of AVG is within ±4 μm.

### 4.3. Calibration

**Figure 7:**
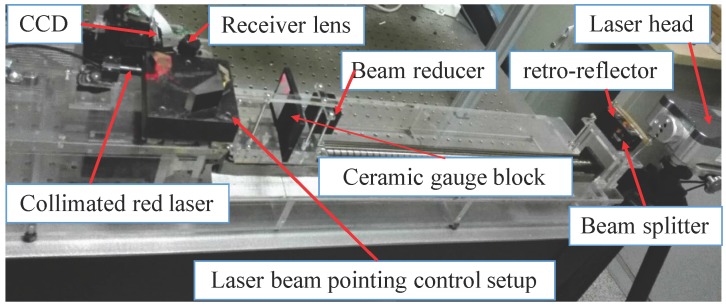
Calibration setup for reduction of the influence of laser beam directional dithering in a laser triangulation displacement probe.

**Figure 8:**
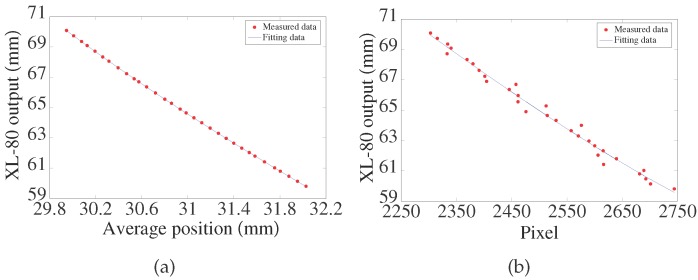
(**a**) Calibration results with laser beam pointing control; (**b**) Calibration results without laser beam pointing control.

**Figure 9:**
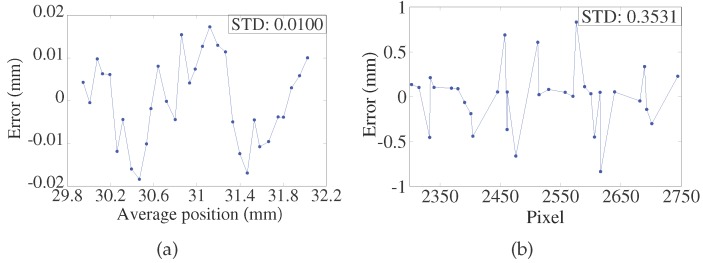
(**a**) Fitting errors with laser beam pointing control; (**b**) Fitting errors without laser beam pointing control.

The LTDP with the LPC was calibrated with a RENISHAW XL-80 (Renishaw plc, Gloucestershire, UK) laser interferometer. The linear resolution of the interferometer is 1 nm. The calibration setup is shown in Figure 7. $P_{2}$ is chosen for comparison of the results. The relative positions of the LTDP with the LPC were calibrated. The criteria ceramic gauge block was driven by a stepper motor point-by-point along the optical axis of the source laser beam with an increment of 0.2 mm within 10 mm. At each point, the collimated red laser was rotated with an increment of 0.2 ° within ±1.1 °. Here, AVG is used as the calibration criterion for the LTDP with the LPC, and the pixel value of $P_{2}$ is used for the calibration criterion of the LTDP without the LPC. The calibration test results are shown in Figure 8. Figure 8a shows the output of the system with the LPC, and Figure 8b shows the output without the LPC. Figure 9a,b shows the fitting errors for each case.

The results show that the calibration curve of the LTDP with the LPC is coincident with that of the laser interferometer. Moreover, the estimated standard deviation (STD) of the probe is shown in Figure 9. The STD is found to be 0.0100 mm with the LPC, as shown in Figure 9. In comparison, the STD is found to be 0.3531 mm without the LPC, as shown in Figure 9.

### 4.4. Repeatability Test

**Figure 10:**
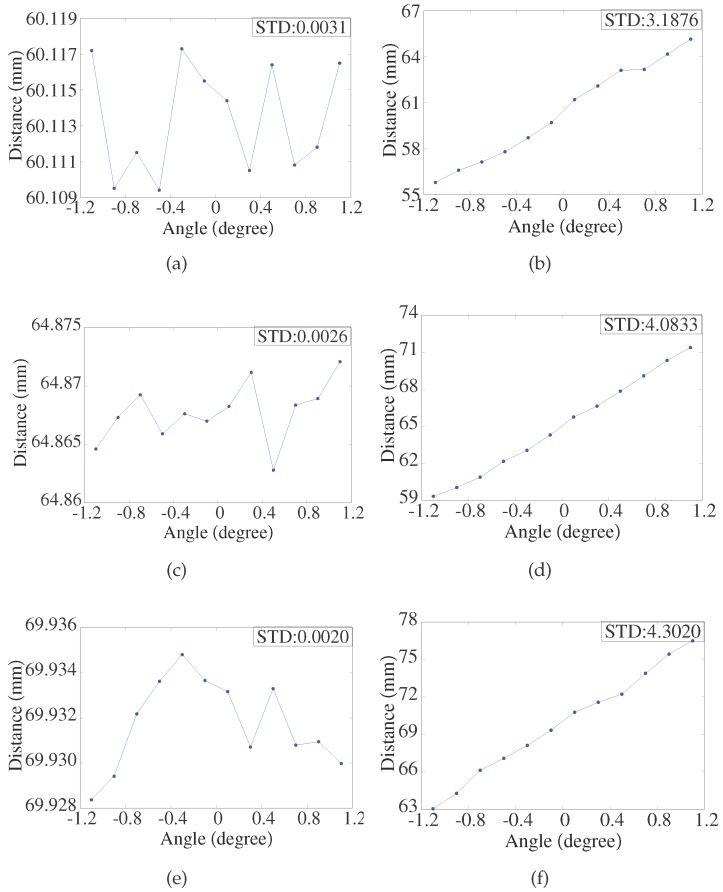
(**a**)Repeatability with laser beam pointing control when the object is located at a closer point; (**b**) Repeatability without laser beam pointing control when the object is located at a closer point; (**c**) Repeatability with laser beam pointing control when the object is located at zero point; (**d**) Repeatability without laser beam pointing control when the object is located at zero point; (**e**) Repeatability with laser beam pointing control when the object is located at a farther point; (**f**) Repeatability without laser beam pointing control when the object is located at a farther point.

As shown in Figure 3, the ceramic gauge block is fixed at a closer point, zero point, and a farther point. The repeatability of the results with and without the LPC is shown in Figure 10 as the collimated laser is rotated within ±1.1° with an increment of 0.2°. As shown in Figure 10, with the LPC, the repeatability accuracy is within ±5 μm and the STD is within 0.0035 mm. In comparison, without the LPC, the repeatability accuracy is ±7 mm and the STD is more than 3 mm.

### 4.5. Nonlinearity Test

**Figure 11:**
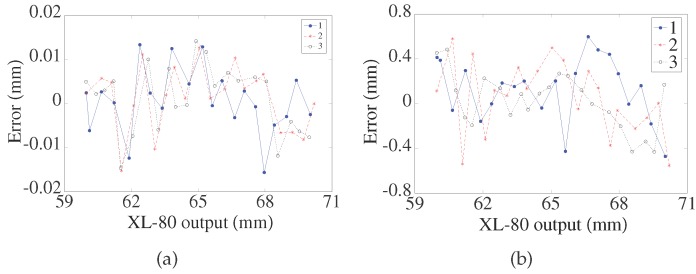
(**a**) Nonlinearity with laser beam pointing control; (**b**) Nonlinearity without laser beam pointing control.

The nonlinearity is expressed as $\left(x_{t}-x_{r}\right)/l_{r}$, where $x_{t}$ is the tested value of the LTDP, $x_{r}$ is the tested value of the XL-80 interferometer (Renishaw plc, Gloucestershire, UK), and $l_{r}$ is the tested range. In this experiment, measurements were performed by moving objects from a closer point to a farther point with an increment of 0.2 mm for three runs. The error between the LTDP and the XL-80 interferometer is shown in Figure 11. As the results show, the nonlinearity with the LPC is within ±0.16 % FS. In comparison, the nonlinearity without the LPC is within ±6 % FS.

## 5. Conclusions

Laser beam dithering is considered as one of the major error sources in LTDP measurements. A theoretical analysis shows that the measurement error will increase approximately linearly as the drift angle increases. A scheme for reduction of the influence of laser beam directional dithering in an LTDP has been developed. This probe consists of a collimated red laser, an LPC, a receiver lens, and a CCD. The collimated red laser beam is split into two symmetrical laser beams by the LPC. Therefore, at the angle at which the laser is dithered, the positional average of the two laser spots on the measured object remains constant. The experimental tests were verified with a dual-beam laser interferometer within the measurement range of 10 mm. With laser beam pointing control, the STD of repeatability of displacement measurement is better than 0.0031 mm, and the nonlinearity is better than ±0.16 %FS. In comparison, without laser beam pointing control, the STD of repeatability of displacement measurement is more than 3 mm, and the nonlinearity is ±6 %FS.

However, the detector selection is limited by the distance between the two points on the detector. Further improvement in the structure of the LPC will result in further improvement in the suitability of the LTDP. Nevertheless, with the current geometrical structure, this system is significantly beneficial to the LTDP. 

## Figures and Tables

**Table 1:** Devices.

Device	Manufacturer	Type	Major Parameters ${}^{1}$
CCD	Toshiba	TCD2566BFG	Resolution 5.25 μm
Ceramic gauge block	Seeman	WB02	Size 50×32×4
Red collimated laser	/	KYL635N10-X1240	λ 635nm
Right-angle prism	Fuyu Optics	/	Size 10×10×10
Pentaprism	XJT	WJ-151515	Size 15×15×10
Half-pentaprism	Daheng Optics	/	Size 30×30×32.6
Rhombic prism	Union Optic	RBP0010	Size 10×10×14.1
Beam splitter	/	/	Size 10×2×30
Receiver lens	/	/	Focus 26.054
Rotational devices	Thorlabs	PRM/M	/

${}^{1}$ All lengths have units of millimeters.
